# Health Care Challenges in the Management of Primary Aldosteronism in Southeast Asia

**DOI:** 10.1210/clinem/dgae039

**Published:** 2024-01-23

**Authors:** Norlela Sukor, Sarat Sunthornyothin, Thang V Tran, Tri Juli Tarigan, Leilani B Mercado-Asis, Satha Sum, Moe Wint Aung, Alice M L Yong, Tania Tedjo, Michael Villa, Nang Ei Ei Khaing, Elena Aisha Azizan, Waye Hann Kang, Vivien Lim, Ada E D Teo, Meifen Zhang, Hieu Tran, Troy H Puar

**Affiliations:** Department of Medicine, Faculty of Medicine, Universiti Kebangsaan Malaysia, Kuala Lumpur 50300, Malaysia; Department of Medicine, Hospital Canselor Tuanku Muhriz, Kuala Lumpur 56000, Malaysia; Department of Medicine, Faculty of Medicine, Chulalongkorn University, Bangkok 10330, Thailand; Department of Endocrinology, University of Medicine and Pharmacy at Ho Chi Minh City, Ho Chi Minh City 700000, Vietnam; Division of Endocrinology, Department of Internal Medicine, Faculty of Medicine, University of Indonesia, Cipto Mangunkusumo Hospital, Jakarta 10430, Indonesia; Faculty of Medicine and Surgery, University of Santo Tomas, Manila 1008, Philippines; Department of Internal Medicine, Calmette Hospital, Phnom Penh 12201, Cambodia; Department of Endocrinology, University of Medicine 1, Yangon General Hospital, Yangon 11131, Myanmar; Department of Internal Medicine, R.I.P.A.S. Hospital, Bandar Seri Begawan BA1712, Brunei Darussalam; Department of Internal Medicine, Faculty of Medicine, Universitas Diponegoro, Semarang, Jawa Tengah 50275, Indonesia; Philippines Center for Diabetes, Thyroid and Endocrine Disorders, St. Luke's Medical Center, Taguig 1634, Philippines; Health Services Research, Changi General Hospital, Singapore 529889, Singapore; Department of Medicine, Faculty of Medicine, Universiti Kebangsaan Malaysia, Kuala Lumpur 50300, Malaysia; Department of Medicine, Hospital Canselor Tuanku Muhriz, Kuala Lumpur 56000, Malaysia; Department of Medicine, Faculty of Medicine and Health Sciences, University Tunku Abdul Rahman, Kuala Lumpur 53300, Malaysia; Gleneagles Medical Centre, Singapore 258500, Singapore; Department of Medicine, Division of Endocrinology, National University Health System, Singapore 119228, Singapore; Department of Endocrinology, Changi General Hospital, Singapore 529889, Singapore; Department of Endocrinology, University of Medicine and Pharmacy at Ho Chi Minh City, Ho Chi Minh City 700000, Vietnam; Department of Endocrinology, Changi General Hospital, Singapore 529889, Singapore; Duke National University of Singapore (NUS) Medical School, Singapore 169857, Singapore

**Keywords:** hyperaldosteronism epidemiology, adrenalectomy, adrenal vein sampling, global health, health care economics, health disparities

## Abstract

**Context:**

While guidelines have been formulated for the management of primary aldosteronism (PA), following these recommendations may be challenging in developing countries with limited health care access.

**Objective:**

We aimed to assess the availability and affordability of health care resources for managing PA in the Association of Southeast Asian Nations (ASEAN) region, which includes low-middle-income countries.

**Methods:**

We instituted a questionnaire-based survey to specialists managing PA, assessing the availability and affordability of investigations and treatment. Population and income status data were taken from the national census and registries.

**Results:**

Nine ASEAN country members (48 respondents) participated. While screening with aldosterone-renin ratio is performed in all countries, confirmatory testing is routinely performed in only 6 countries due to lack of facilities and local assays, and cost constraint. Assays are locally available in only 4 countries, and some centers have a test turnaround time exceeding 3 weeks. In 7 countries (combined population of 442 million), adrenal vein sampling (AVS) is not routinely performed due to insufficient radiological facilities or trained personnel, and cost constraint. Most patients have access to adrenalectomy and medications. In 6 countries, the cost of AVS and adrenalectomy combined is more than 30% of its annual gross domestic product per capita. While most patients had access to spironolactone, it was not universally affordable.

**Conclusion:**

Large populations currently do not have access to the health care resources required for the optimal management of PA. Greater efforts are required to improve health care access and affordability. Future guideline revisions for PA may need to consider these limitations.

Hypertension affects 1 in 4 adults worldwide, of whom 5% to 20% have a treatable condition: primary aldosteronism (PA) (1). Patients with PA have a 2- to 3-fold greater risk of cardiovascular events, renal failure, and poorer quality of life (2, 3). In addition, patients with unilateral PA can potentially be cured of hypertension with adrenalectomy. However, recent studies have shown that less than 2% of at-risk patients are ever screened (4, 5). These estimates may be even lower in settings with limited resources, especially in lower-middle-income countries (LMICs).

Endocrine Society guidelines for the management of PA recommend various tests (screening, confirmatory, and subtyping) before decision on the treatment (mineralocorticoid-receptor antagonists [MRAs] or unilateral adrenalectomy) (6). However, biochemical tests for aldosterone and renin are not widely available in most routine clinical laboratories. To subtype PA, computed tomography (CT) is often inaccurate, and adrenal vein sampling (AVS) is recommended in most patients seeking curative surgery (7). However, AVS is technically challenging, and even in specialized centers, the overall success rates are about 80% (8). In LMICs with limited medical resources, there is a common prioritization of emergency services over the establishing of medical care for less emergent conditions, such as PA (9). Hence, it is likely that elective procedures, such as AVS and adrenalectomy, are given less priority. Even if these health care facilities were available, patients may not be able to afford these investigations or treatment. Hence, we set out to assess the availability and affordability of health care resources that are necessary for the optimal management of PA, in the Association of Southeast Asian Nations (ASEAN) region.

## Materials and Methods

This study is an international, multicenter, cross-sectional questionnaire-based survey of specialists managing PA. Nine countries in the ASEAN region were included. Indonesia, the Philippines, Vietnam, Myanmar, and Cambodia are designated as lower-middle-income countries (LMICs), Malaysia and Thailand as upper-middle-income countries (UMICs), and Singapore and Brunei as high-income countries, according to World Bank classification (10). During this study, the ASEAN Network of Adrenal Hypertension was formed. The study was approved by the ethics review committee who waived requirement for informed consent (SingHealth Centralised Institutional Review Board, reference: 2022/2672). The first 2 meetings involved open-ended discussions to explore the challenges faced by each country. Subsequently, a questionnaire was drafted and finalized (Supplementary appendix) (11), to assess the (1) availability of health care resources, and (2) affordability for patients managed for PA in the ASEAN region.

Each country representative distributed the questionnaire to colleagues in their respective countries. Respondents were all from different centers (private and/or public), and often different regions, to provide a better sampling of the entire country. For affordability, respondents were asked to estimate the out-of-pocket costs for their patients, and the proportion of their patients who were able to afford the tests and treatment.

National data were obtained from national registries. The out-of-pocket costs for tests and treatment were compared to national data on gross domestic product (GDP) per capita obtained from World Bank (10). Conversion of each local currency to US dollars was performed using the US government treasury converter (https://fiscaldata.treasury.gov/currency-exchange-rates-converter/) (Table 1). The population of adults (aged 30-79 years) in each country was taken from United Nations data (12). We estimated the population of patients with PA in each country by using national data of the adult population, and local prevalence of hypertension from a prior study in this region (which was 5% among patients with hypertension in primary care) (13, 14).

**Table 1.** Affordability and out-of-pocket costs to patients in Southeast Asia

Country		Myanmar	Cambodia	Philippines	Vietnam	Indonesia	Thailand	Malaysia	Brunei	Singapore
World Bank economy classification		LMIC	LMIC	LMIC	LMIC	LMIC	UMIC	UMIC	HIC	HIC
No. of centers surveyed		4	4	7	9	7	6	7	1	4
Sector (public/private/both)		1/0/3	3/0/1	0/4/2	9/0/0	7/0/2	4/0/2	5/2/0	1/0/0	3/1/0
**Patients who can afford investigations and treatment, %**										
Screening	ARR	50 (40-100)	50 (30-100)	55 (50-100)	100 (100-100)	25 (0-90)	95 (80-100)	100 (50-100)	95	100 (99-100)
Confirmatory*^a^*		45 (30-50)	0 (0-50)	50 (10-100)	100 (70-100)	38 (0-80)	90 (20-100)	100 (10-100)	95	100 (90-100)
Subtyping	CT imaging	100 (80-100)	80 (50-100)	83 (50-100)	100 (50-100)	100 (25-100)	100 (80-100)	100 (80-100)	95	100 (99-100)
	AVS	0	0	8 (0-75)	0 (2-70)	13 (0-50)	80 (70-100)	93 (20-100)	NA	93 (75-99)
Treatment	Adrenalectomy	80 (30-100)	60 (50-100)	63 (0-100)	80 (60-100)	95 (25-100)	95 (80-100)	90 (20-100)	95	93 (80-99)
	Medications	95 (70-100)	98 (80-100)	100 (80-100)	100 (80-100)	100 (25-100)	100 (20-100)	100 (60-100)	100	100 (99-100)
**Out-of-pocket costs (in US dollars)**										
Screening	ARR	119 (80-190)	102 (100-104)	126 (45-180)	25 (0-51)	225	43 (0-116)	1 (0-114)	0.75	37 (37-149)
Confirmatory*^a^*		95 (61-95)	40 (0-80)	1439 (90-360)	25 (4-64)	129	58 (0-145)	11 (0-455)	0	299 (112-373)
Subtyping	CT imaging	131 (95-190)	165 (150-200)	162 (72-225)	64 (42-127)	97	145 (0-579)	1 (0-341)	0	299 (149-373)
	AVS	NA	NA	1439 (899-2698)	339 (127-339)	515	536 (0-2897)	227 (1-6818)	0	746 (373-3731)
Treatment	Adrenalectomy	476 (476-952)	1200 (1000-1500)	1798 (1349-3597)	847 (212-1694)	2061	2897 (0-8691)	455 (0-4545)	0	1493 (1119-18 657)
GDP per capita (World Bank data)		1210	1625	3461	3757	4333	7066	11 109	31 449	72 794
Median cost of (AVS + adrenalectomy)/GDP, (%)		39.3	73.8	93.5	31.6	59.5	48.6	6.1	0.0	3.1

Results are median (minimum to maximum).

Abbreviations: ARR, aldosterone-renin-ratio; AVS, adrenal vein sampling; CT, computed tomography; GDP, gross domestic product; HIC, high-income country; LMIC, lower-middle-income country; NA, not applicable; UMIC, upper-middle-income country.

^*a*^Confirmatory tests used were intravenous saline-loading test, oral saline loading test, fludrocortisone suppression test, or captopril challenge test.

## Results

Specialists from 9 countries, comprising 48 respondents in the ASEAN region, responded (see Table 1): 30 in public centers (teaching or nonteaching), 8 in private practice, and 10 working both in public and private practice. Forty-seven (98%) were endocrinologists, with one internal medicine physician. The reasons for patients being referred for suspected PA included hypertension with hypokalemia (n = 49), hypertension with adrenal incidentaloma (n = 38), young-onset hypertension (n = 33), drug-resistant hypertension (n = 30), and severe hypertension (n = 15).

### Screening Tests

Most respondents “always” or “often” performed a screening aldosterone-renin ratio (ARR) in patients with suspected PA. However, 10 respondents did not perform this routinely (Indonesia, n = 4; Philippines, n = 4; Cambodia, n = 2). The reasons cited were that patients could not afford it (n = 10) or the assays were unavailable locally and turnaround time was too long (n = 5). When not performed, some respondents would treat patients with MRA. Aldosterone and renin assays were performed locally in 4 countries (Philippines, Vietnam, Thailand, and Malaysia). In the remaining countries, assays were sent overseas for analysis to neighboring countries (Thailand and Malaysia) or the United States (Mayo Laboratory). Assay turnaround time varied between centers: within the day (n = 2), within 1 week (n = 21), within 3 weeks (n = 17), and greater than 3 weeks (n = 7).

### Confirmatory Tests

**Figure 1.**
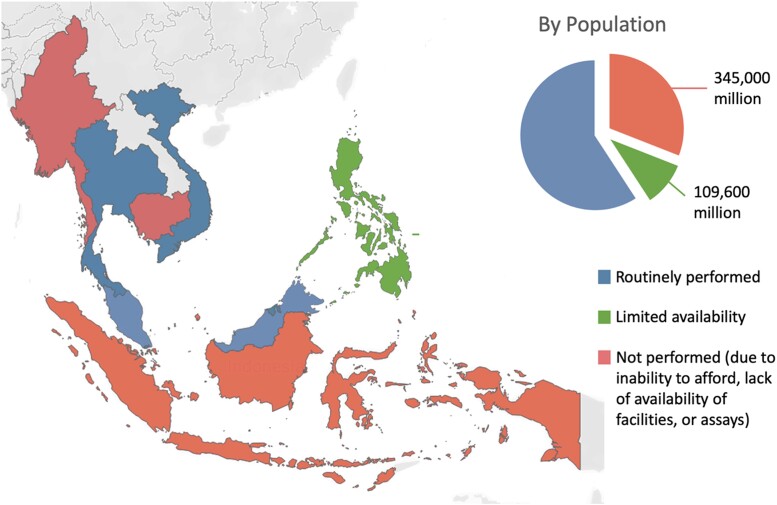
Access to confirmatory testing in ASEAN (Association of Southeast Asian Nations).

In 5 countries (Vietnam, Thailand, Malaysia, Singapore, and Brunei), confirmatory tests were “always” or “often” performed (Fig. 1). Confirmatory tests were omitted in patients with overt PA (markedly elevated aldosterone level, suppressed renin, and spontaneous hypokalemia). Conversely, in 4 countries (Indonesia, Philippines, Myanmar, and Cambodia), confirmatory tests were performed “occasionally,” “seldom,” or “never.” The reasons for omission were patients could not afford it (n = 8), assays were not available or took too long (n = 8), or there was a lack of facilities to perform the confirmatory tests (n = 6). The most common first-line confirmatory test was intravenous saline infusion (n = 41). Other tests included oral saline loading test (n = 3), fludrocortisone suppression test (n = 1), and captopril challenge test (n = 1). When confirmatory tests were omitted, respondents would assume the patient had PA, with 7 respondents proceeding with subtype tests (and surgery if unilateral PA), while 4 would proceed only to MRA.

### Subtype Tests

**Figure 2.**
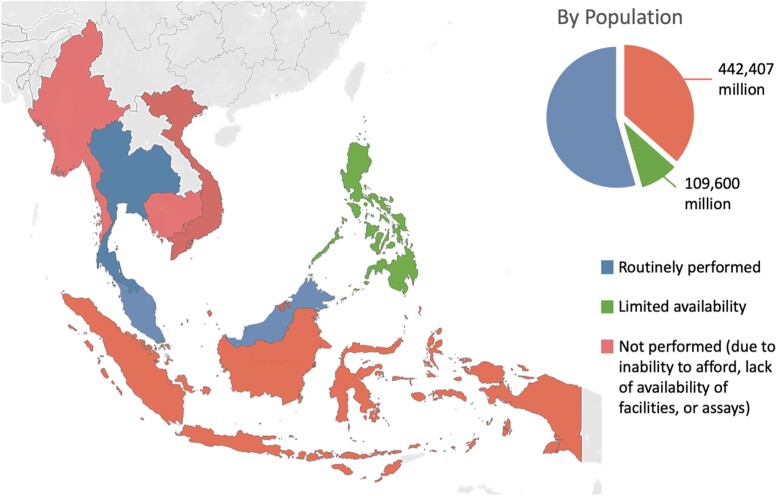
Access to adrenal vein sampling in ASEAN (Association of Southeast Asian Nations).

Almost all respondents “always” or “often” performed CT imaging in patients with PA. Two respondents in Indonesia “seldom” performed CT imaging because their patients could not afford it. In 47 of 48 centers, CT imaging was performed at the same center. Three countries (Thailand, Malaysia, and Singapore) “always,” “often,” or “occasionally” performed AVS. When not performed, the main reasons were that patients were not keen (or suitable) for adrenalectomy (n = 11), AVS was not required (n = 4), or AVS success rates were poor (n = 4). Conversely, AVS was “seldom” or “never” performed in the remaining countries (Fig. 2) (Indonesia, the Philippines, Vietnam, Myanmar, Cambodia, and Brunei), with 2 exceptions: 1 center in the Philippines “often” and 1 in Vietnam “occasionally” performed AVS. The reasons cited for not performing AVS in these countries were different: patients could not afford it (n = 14), there was a lack of facilities such as fluoroscopy suites (n = 17), and there were no trained personnel to perform AVS (or poor AVS success rates) (n = 28). When AVS was not available, respondents chose MRA treatment only (n = 5), offered surgery using CT findings (n = 9), or offered both options to patients (n = 11).

### Treatment

Most respondents “always” or “often” discussed the option of adrenalectomy unless the patient was a poor surgical candidate. In Indonesia (n = 2) and Cambodia (n = 1), respondents “occasionally” or “seldom” discussed adrenalectomy because there was a lack of operating theaters and/or AVS, or patients could not afford it. Adrenalectomies were performed at their centers (n = 40), at another center (<1 hour away) (n = 4), or at another center (>1 hour away) (n = 4). Spironolactone was available at all centers. Eplerenone was available at only some centers in Indonesia (n = 1), Vietnam (n = 1), Myanmar (n = 4), Cambodia (n = 1), Malaysia (n = 3), and Singapore (n = 4), while amiloride was available only in Malaysia (n = 1) and Singapore (n = 3).

### Affordability

Respondents from 6 countries reported that 80% or less of their patients could afford a screening test: Indonesia (n = 5), Cambodia (n = 3), Myanmar (n = 3), Malaysia (n = 2), Philippines (n = 4), and Thailand (n = 1) (see Table 1). For confirmatory tests, most patients in Indonesia, Myanmar, and Cambodia could not afford this, while in the Philippines, 50% (range, 10%-100%) of patients could afford it. Respondents reported that the majority of patients could afford CT imaging and adrenalectomy. However, AVS was either unavailable or not affordable for most patients in 5 countries (Indonesia, the Philippines, Vietnam, Myanmar, and Cambodia). One respondent from each of these countries (Indonesia, the Philippines, Vietnam, Myanmar, Cambodia, Thailand, and Malaysia), reported that 80% or less of their patients could afford spironolactone treatment.

The out-of-pocket costs for investigations and treatment varied widely between countries (see Table 1). In Brunei, due to 100% national health care coverage for local citizens, the management of PA is almost entirely free. In other countries, the out-of-pocket cost of AVS varies from USD 227 in Malaysia, to USD 1439 in the Philippines, while the out-of-pocket cost of adrenalectomy varies from USD 455 in Malaysia, to USD 1798 in the Philippines. When considered as a percentage of GDP per capita, the combined cost of AVS and adrenalectomy was highest in the Philippines, amounting to 93.5%, followed by Cambodia (73.8%), and Indonesia (59.5%). In 6 countries, the combined cost of AVS and adrenalectomy was more than 30% of the annual GDP per capita.

### Number of Patients With Primary Aldosteronism Diagnosed and Treated

Most centers in the ASEAN region had 5 to 100 patients referred for suspected PA per year, with 3 to 30 patients diagnosed with PA (Table 2). Most centers either do not offer AVS or have fewer than 10 patients per year undergoing AVS, with the exception of several centers in Thailand, Malaysia, and Singapore. These centers correspondingly have more patients undergoing adrenalectomy. Centers in Vietnam have the highest volume of patients undergoing adrenalectomy, with surgery guided by CT imaging since AVS is largely unavailable.

**Table 2.** Number of patients with primary aldosteronism presenting to centers in the Association of Southeast Asian Nations region each year

Country	Myanmar	Cambodia	Philippines	Vietnam	Indonesia	Thailand	Malaysia	Brunei	Singapore
No. of centers surveyed	4	4	7	9	7	6	7	1	4
New cases with suspected PA per center	9 (5-24)	8 (5-20)	10 (8-20)	35 (20-200)	5 (5-13)	50 (30-200)	40 (5-120)	20	100 (100-100)
New cases with confirmed PA per center	7 (2-16)	2 (0-5)	5 (1-5)	20 (10-40)	3 (0-9)	28 (5-45)	5 (0-15)	6	30 (10-30)
Total new cases with confirmed PA in all centers	31	13	19	180	27	155	37	6	70
Patients undergoing AVS per centers within the country	0	0	0 (0-5)	1 (0-5)	0 (0-1)	25 (1-36)	2 (0-12)	0	10 (8-18)
Patients with PA undergoing adrenalectomy per center	7 (1-15)	2 (0-5)	2 (0-5)	17 (7-35)	1 (0-8)	10 (2-15)	2 (0-8)	4	5 (3-10)
Total patients with PA undergoing adrenalectomy in all centers	29	9	13	139	7	54	27	4	18
Population of adults (aged 30-79 y)*^a^*	25 876 504	7 390 631	46 796 331	50 523 151	137 216 836	40 837 968	15 689 115	225 838	2 557 671
Prevalence of hypertension (%)*^a^*	37.60	25.60	33.75	29.65	40.20	29.15	40.75	46.3	31.25
Expected population of patients with PA*^b^*	486 478	94 600	789 688	749 006	2 758 058	595 213	319 666	5228	39 964

Results are median (minimum to maximum).

Abbreviations: AVS, adrenal vein sampling; PA, primary aldosteronism.

^*a*^Data were obtained from population-representative studies (12).

^*b*^Assumption based on population of adults × prevalence of hypertension × prevalence of PA (set at 5%) (13).

The number of patients with PA diagnosed was well below the estimated number in all countries. For instance, in Cambodia, there are only 4 major centers nationwide providing endocrine services. When all 4 centers were surveyed, a total of 13 patients with PA were being diagnosed each year, well below the expected number of 93 861 patients with PA in Cambodia.

## Discussion

In our international Southeast Asia study, we report a lack of availability and affordability of investigations and treatment required for the optimal management of patients with PA. Despite PA being a highly prevalent and curable cause of hypertension, assays for aldosterone and renin required for its diagnosis are not widely available in this region. For half of the ASEAN population (∼0.5 billion individuals), those suspected to have PA will not be sent for confirmatory tests or subtyping with AVS, as currently recommended by Endocrine Society guidelines (6). This is due to the lack of the necessary health care infrastructure and/or personnel, and the lack of affordability for patients, many of whom live in LMICs.

Screening tests for aldosterone and renin levels is the minimal prerequisite for diagnosis of PA. However, most countries in ASEAN rely on overseas laboratories for assays. This has many consequences. First, this increases test turnaround time, which is 3 weeks or more at many centers. Second, this contributes to an increased diagnostic interval both for confirmatory tests and AVS, which require biochemical results. Third, even where available, screening tests with ARR are frequently performed only by endocrine specialists. Since most patients with PA have hypertension and are often asymptomatic, screening should be performed at primary care. Hence, many patients with PA are left undiagnosed. Despite guideline recommendations for high-risk hypertensive patients to be screened, recent studies have reported worldwide screening rates of less than 2% in the community (4), and 3.4% in a tertiary center with PA expertise (15). In a Scandinavian study, the investigators found a lower-than-expected prevalence of PA at below 0.022% (16). In this study, we surveyed PA experts from major tertiary centers in each country. Based on our estimates, less than 0.01% of all patients with PA are ever diagnosed in the ASEAN region.

Both confirmatory testing and AVS for subtyping are cornerstones in the recommended workup of patients with PA (6). However, confirmatory testing is routinely omitted in 4 countries, while AVS is not available in 5 countries with an estimated 0.5 billion population –– 80% of the ASEAN population, and 9% of the world's population. When omitting confirmatory tests and AVS, physicians either chose to start empirical MRA therapy, or assumed these patients had PA and proceeded to offer adrenalectomy based on CT findings. This can lead to either fewer patients being offered curative surgery, or some patients undergoing unnecessary and incorrect adrenalectomy. While confirmatory testing is recommended for the majority of patients, it may be omitted in those with overt and severe hyperaldosteronism (17). However, there have also been arguments that confirmatory testing may be inaccurate, and wrongly exclude patients with PA (18).

There were several reasons cited for the omission of AVS in ASEAN countries. First, a lack of an interventionalist with the necessary expertise. Even in developed countries, successful cannulation is achieved in only approximately 80% of procedures (8). In our study, the reported success rates were less than 50% in several countries, which is expected as these centers performed fewer than 5 procedures per year. Second, several countries lack the necessary infrastructure such as fluoroscopy rooms or specialized catheters for vein sampling. Third, the cost of AVS was too prohibitive. With the exception of young patients with a clear unilateral adenoma, the majority of patients with PA are recommended to undergo AVS (6). While a randomized controlled trial found similar blood pressure outcomes both with a CT-guided vs AVS-guided approach, this study was underpowered to evaluate for biochemical cure of PA (19). A subsequent multicenter retrospective study reported better biochemical outcomes with an AVS-guided, compared to CT-guided, approach (20). Hence, this reiterates the importance of AVS for subtyping of PA.

The lack of affordability is a major concern in the ASEAN region. Screening tests, where available, were occasionally not affordable, mainly in the LMICs. The requirement to send these tests overseas may have added to the increased cost. Without screening tests and a diagnosis of PA, patients cannot be offered adrenalectomy. This may explain why a higher proportion of patients could afford adrenalectomy compared to a screening test in Myanmar. Adrenalectomy generally leads to better outcomes compared to medical treatment (21, 22). However, the combined cost of AVS and adrenalectomy can be prohibitive. Some countries may offer more extensive and comprehensive health care than others due to various factors such as political reasons, economic affluence, and population literacy/education. While some countries have close to universal health insurance coverage (Brunei and Malaysia) and treatment is largely affordable, most other countries required a significant proportion of copayment from patients. In 6 countries, these out-of-pocket costs were more than 30% of the annual GDP per capita. As a minimum, patients with PA should be offered treatment with MRAs. However, despite spironolactone being one of the World Health Organization’s essential medications, it was not universally affordable for all patients (23).

Apart from the variability of health care access between countries, we also found considerable variability within countries. There are differences in the availability and costs of health care within each country (eg, between urban vs rural, region-specific, or public vs private health care), leading to differences in health outcomes. This was more evident in larger countries like Indonesia and the Philippines, with more than 25 000 islands combined. In the Philippines, AVS is available only in larger cities like Manila, while in Indonesia, ARR screening is available only in larger cities on the islands of Java and Sumatra. One study highlighted the enormous disparities in health outcomes between different provinces of Indonesia, with western provinces (Java and Sumatra) having a 10-year longer healthy life expectancy compared to the eastern provinces, consistent with our findings (24). Previous studies have shown that people living in urban centers, or near tertiary medical centers, were more likely to be screened for PA (4, 25). Hence, greater efforts need to be undertaken to increase accessibility for screening, particularly in rural and remote areas. In addition to the challenges we have highlighted, some countries have a substantial proportion of migrant workers (26, 27), or refugees, who have even less health care access. In other countries, such as Myanmar, political situations may also affect health care accessibility (28).

Our study highlights important barriers regarding managing PA in this understudied ASEAN population, with the majority living in LMICs. Similar issues may face other countries in South Asia, Africa, and South America. There are several ways to address these issues. First, we need to improve the availability of aldosterone and renin assays. After this study's completion, these assays were made available in Singapore (29). However, this remains a priority for many other countries worldwide. Second, screening by primary care physicians should be more accessible as well as affordable to patients through subsidies. Screening for PA in primary care has been shown to be cost-effective (30), but cost-effectiveness depends on local economic and health care factors that need to be determined for each country. Third, building infrastructure and the training of a multidisciplinary team of health care professionals to manage PA is essential.

Until these endeavors are achieved, we should consider how patients with PA will be best managed. Current international guideline recommendations are based on the best clinical evidence from studies in developed countries. It may be useful to consider the limitations faced by LMICs in future guideline iterations. As aldosterone and renin assays are still not widely available, many patients may have an opportunity for only a single positive screening test before proceeding with subsequent localization or treatment. Hence, an option to omit confirmatory tests in more cases of overt PA may be helpful. Even in centers with AVS facilities, CT-guided adrenalectomy is being performed when AVS is inconclusive, with similar blood pressure outcomes compared to an AVS-guided approach (20). Hence, where AVS is not available, offering adrenalectomy based on CT may be an option for some health care providers. In the future, functional imaging or measurement of hybrid hormones may obviate the necessity for AVS expertise, but these may not be widely available to LMICs.

The strengths of our study include being the first multinational study covering mainly LMICs, focusing on the accessibility and affordability of treating PA. We have a wide representation of respondents who work both in public and private health care facilities, and manage a combined population of 600 million or 10% of the world's population. We recognize several limitations of our study. First, most of our data were obtained from clinicians working in major referral centers, which may lead to a selection bias. However, we aimed to assess the availability of facilities in each country, and the lack of such facilities in these tertiary centers would demonstrate a gap in the country at large. Second, there may be a selection bias of patients presenting to these centers for medical care. Patients who live too far from these centers or lack the financial support to travel and seek care are more likely to be excluded. Hence, our estimates for the affordability of health care are likely an underestimate, and even more patients in the ASEAN region face cost constraints. Third, this study covers only the ASEAN region (10% of the world population). Other LMICs in Africa, South Asia, and South America likely face similar challenges in treating PA. Fourth, this is a questionnaire-based survey, and estimates (eg, case load, affordability, out-of-pocket costs) may be prone to recall bias. However, obtaining patient-level data in many of these LMICs is not currently possible, and this forms the best available data. Finally, our estimates of the prevalence of PA are based on only one prevalence study, which found a prevalence of PA of 5% in the primary care in Singapore (13). Even at our conservative estimates, a large proportion of patients in the ASEAN region are undiagnosed. This also highlights the need for more studies in these countries.

In conclusion, we highlight that the management of PA in the ASEAN region is fraught with many challenges, such as a need for more facilities for testing, and the cost limitations of the population. It may be helpful for future international guidelines to offer alternative solutions in managing PA when resources are limited. With limited resources for managing PA in LMICs, the priority may be to target patients with PA with the most overt disease, and who are most likely to benefit from intervention.

## Data Availability

Original data generated and analyzed during this study are included in this published article.

## Abbreviations

ARR: aldosterone-renin ratio
ASEAN: Association of Southeast Asian Nations
AVS: adrenal vein sampling
CT: computed tomography
GDP: gross domestic product
LMICs: lower-middle-income countries
MRAs: mineralocorticoid-receptor antagonists
PA: primary aldosteronism
UMICs: upper-middle-income countries
